# SIRT1-metabolite binding histone macroH2A1.1 protects hepatocytes against lipid accumulation

**DOI:** 10.18632/aging.100632

**Published:** 2014-01-28

**Authors:** Valerio Pazienza, Michela Borghesan, Tommaso Mazza, Fareeba Sheedfar, Concetta Panebianco, Roger Williams, Gianluigi Mazzoccoli, Angelo Andriulli, Tomoko Nakanishi, Manlio Vinciguerra

**Affiliations:** ^1^ Department of Medical Sciences, Gastroenterology Unit, IRCCS “Casa Sollievo della Sofferenza” Hospital, San Giovanni Rotondo, Italy; ^2^ Department of Medical Sciences, Division of Internal Medicine, IRCCS “Casa Sollievo della Sofferenza” Hospital, San Giovanni Rotondo, Italy; ^3^ Bioinformatics Unit, IRCCS “Casa Sollievo della Sofferenza” - Mendel Laboratory, Rome, Italy; ^4^ University of Groningen, University Medical Center Groningen (UMCG), Molecular Genetics, Groningen, The Netherlands; ^5^ Institute of Hepatology, Foundation for Liver Research, London, UK; ^6^ Division of Molecular Biology, School of Life Sciences, Faculty of Medicine, Tottori University, Yonago, Tottori, Japan; ^7^ University College London (UCL) – Institute for Liver and Digestive Health, Division of Medicine, Royal Free Hospital, London, UK; ^8^ Euro-Mediterranean Institute of Science and Technology (IEMEST), Palermo, Italy

**Keywords:** histone macroH2A1, hepatocyte, lipids, gene expression

## Abstract

Non-alcoholic-fatty-liver-disease (NAFLD) encompasses conditions associated to fat deposition in the liver, which are generally deteriorated during the aging process. MacroH2A1, a variant of histone H2A, is a key transcriptional regulator involved in tumorigenic processes and cell senescence, and featuring two alternatively splicing isoforms, macroH2A1.1 and macroH2A1.2. MacroH2A1.1 binds with high affinity O-acetyl ADP ribose, a small metabolite produced by the reaction catalysed by NAD+-dependent deacetylase SIRT1, whereas macroH2A1.2 is unable to do so. The functional significance of this binding is unknown. We previously reported that the hepatic levels of macroH2A1.1 and macroH2A1.2 are differentially expressed in mice models of NAFLD. Here we show that over-expression of macroH2A1.1, but not of macroH2A1.2, is able to protect hepatocytes against lipid accumulation. MacroH2A1.1 over-expressing cells display ameliorated glucose metabolism, reduced expression of lipidogenic genes and fatty acids content. SIRT1/macroH2A1.1-dependent epigenetic regulation of lipid metabolism may be relevant to NAFLD development.

## INTRODUCTION

**Figure 1:**
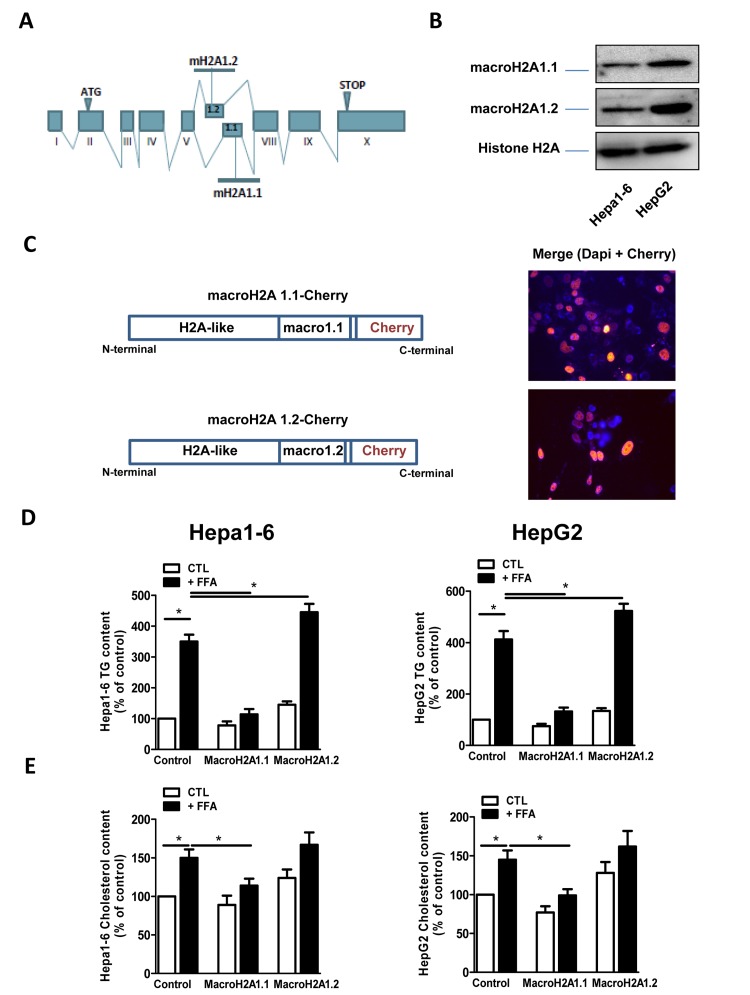
(**A**) Schematic representation of the structure of the macroH2A1 gene, which contains two mutually exclusive exons (macroH2A1.1 and macroH2A1.2). (**B**) Histone lysates were isolated from Hepa1-6 and HepG2 cells and processed for immunoblotting. Representative images for macroH2A1.1, macroH2A1.2 and histone H2A are shown. (**C**) *Left:* schematic representation of the constructs used in this study, composed of the macroH2A1.1 or macroH2A1.2 gene (made of a H2A-like domain and the relative macro domain) fused at the C-terminal to cherry protein. *Right:* Transient over-expression of cherry-tagged macroH2A1.1 or macroH2A1.2 constructs in Hepa1-6 cells. Nuclei were counterstained with DAPI. In the overlay image, transfected cells overexpressing macroH2A1.1 appear in pink/orange. (**D**) and (**E**) Triglyceride and cholesterol content in Hepa1-6 and HepG2 cells cells overexpressing macroH2A1 isoforms. Cells were transfected with either an empty vector (control, CTL) or with Cherry-tagged macroH2A1.1 and macroH2A1.1 constructs. 24 hours later cells were exposed to a 100 mM mixture of FFA, for an additional 24 hours. Triglyceride (**D**) and cholesterol (**E**) content were assayed using commercial kits. Results are expressed as percentage of controls, means ± SEM of four independent experiments. *p<0.05.

The current pandemic in obesity/metabolic syndrome is a risk factor for many types of diseases, including cancer. In up to 90% of cases, obesity is accompanied by non-alcoholic-fatty-liver-disease (NAFLD) [1]. NAFLD is the consequence of an imbalance between lipid availability through fatty acid (FA) uptake and *de novo* lipogenesis, and lipid secretion and disposal via free fatty acid (FFA) oxidation, resulting in hepatic steatosis [2]. In 10% of the cases NAFLD will progress to steatohepatitis (NASH), and in 8-26% to cirrhosis, with an increasing incidence of cases with NAFLD that develop hepatocellular carcinoma (HCC) at an earlier stage [3]. There is substantial evidence that the progression from NAFLD to HCC is accrued by the aging process [4]. Epigenetic mechanisms of nuclear chromatin remodelling, such as DNA methylation, post-translational modifications of histones, and incorporation of histone variants into the chromatin are increasingly recognized as crucial factors in the pathophysiology of NAFLD and in several age-associated diseases [5-7]. In fact, alterations in hepatic metabolism and proliferation during steatosis are triggered by changes in gene transcriptional patterns dependent on the degree of nuclear chromatin compaction. The latter is regulated at several levels, allowing transcriptional plasticity [8]: one way is the replacement of canonical histones around which DNA is wrapped (H2A, H2B, H3 and H4) with the incorporation of histone variants. The histone variant of H2A, known as macroH2A1, is believed to act as a strong transcriptional modulator that can either repress transcription [9, 10], or activate it in response to as yet undefined growth signals [11]. The phenotype of macroH2A1 knock out (KO) mice consists of mild and variable derangements in systemic and/or hepatic glucose and lipid metabolism, depending on the strain [12, 13]. MacroH2A1 is present in 2 isoforms, macroH2A1.1 and macroH2A1.2, which are generated upon RNA alternative exon splicing (Figure 1A). These isoforms differ in just few amino acids, a conserved structural difference that explains why macroH2A1.1 can bind ADP-ribose-like metabolites produced by NAD+-dependent histone deacetylase SIRT1, such as O-acetyl ADP ribose (OAADPR), or by polyADP-ribose polymerase 1 (PARP1), while macroH2A1.2 is unable to do so [14-17]. Interestingly, this binding was the first described direct molecular interaction between intermediate metabolism and the chromatin, whereby a metabolite can impinge on and tweak gene expression [14-16]. MacroH2A1 isoforms regulate cancer cell growth *in vitro* [18] and their expression levels have been shown to mark HCC, colon and lung cancer recurrence [19-21]. MacroH2A1 isoforms accumulate massively in the nuclei of senescent hepatocyte and fibroblasts, although the functional significance of this finding is unknown [19, 22]. Moreover, KO of all macroH2A1 isoforms induced the progression of the malignant phenotype of melanoma through increased expression of CDK8 oncogene [23]. The epigenetic regulation of oncogenes and/or tumor suppressors is particularly relevant during NAFLD and HCC, since the activities of these genes often link mechanistically the two conditions [24-26]. In a recent study, we observed that protein levels of macroH2A1.2, but not macroH2A1.1, are dramatically increased in the liver of the high-fat/diethynitrosamine diet and the genetic liver-specific PTEN knock-out (KO) mice models of NAFLD [21], suggesting a differential functional role for these sister molecules in NAFLD pathogenesis. In this study we explore this hypothesis *in vitro* using two different hepatic cell lines, murine Hepa1-6 and human HepG2. We show that OAADPR-binding macroH2A1.1, but not macroH2A1.2, consistently ameliorates glucose metabolism and protects against lipid accumulation by altering the expression of genes involved in fatty acids (FA) metabolism and the composition of cellular membranes.

## RESULTS

### MacroH2A1.1 protects Hepa1-6 and HepG2 cells from lipid accumulation

**Figure 2:**
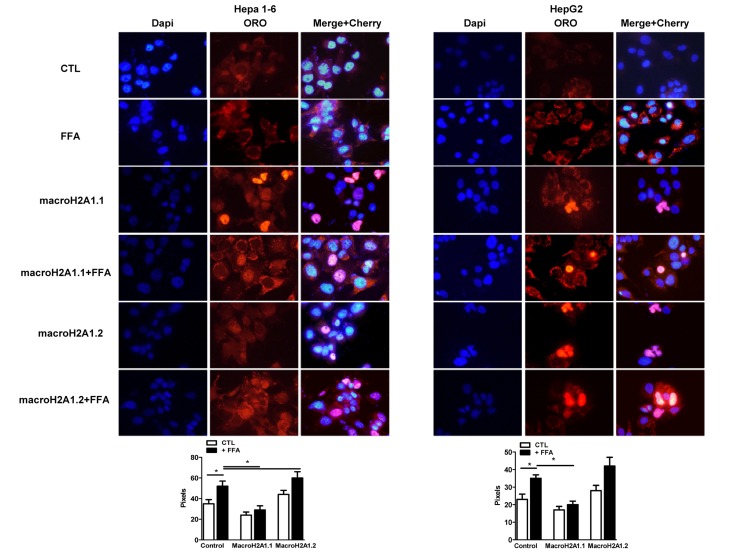
Overexpression of macroH2A1 isoforms (macroH2A1.1 or macroH2A1.2) and lipid accumulation in Hepa1-6 cells and HepG2 cells. Upper panels: cells were transiently transfected with Lipofectamine with either an empty vector (control, CTL) or with Cherry-tagged macroH2A1.1 and macroH2A1.1 constructs. 24 hours post transfection cells were exposed to a 100 mM 1:1 mixture of oleic acid/linoleic acid (FFA) conjugated with albumin, for an additional 24 hours. Cells were then fixed, nuclei stained with DAPI (blue) and lipids with ORO. Overlay of Cherry tagged macroH2A1-transfected nuclei and DAPI staining is observed in pink. Lower panel: quantifications of ORO stained areas are means ± SEM of 1000 cells per condition. *p<0.05.

MacroH2A1.2, but not of macroH2A1.1, is upregulated in the liver of NAFLD *in vivo* models [21]; however the function of these isoforms in NAFLD is unknown. We examined the effect of macroH2A1 isoforms on lipid accumulation in two well established hepatic cell lines, Hepa1-6 and HepG2 cells [26, 27]. HepG2 cells expressed more abundant endogenous levels of macroH2A1.1 and macroH2A1.2 than Hepa1-6 cells. (Figure 1B) However, within each cell line similar endogenous levels of macroH2A1.1 and macroH2A1.2 were expressed (Figure 1B): this not being confounding factor, we ectopically over-expressed one or the other isoform. Transient transfection with cherry-tagged macroH2A1.1 or macroH2A1.2 constructs (Figure 1C, left) yielded a 30-40% efficiency (Figure 1C, right) and did not have any effect on the cell cycle, as measured by the percentage of cells gated in the G0/G1, S and G2/M phases by flow cytometry in Hepa1-6 cells (Table I, n=3). Similar results were obtained in HepG2 cells (*data not shown*). 24 hours post-trasfection Hepa1-6 and HepG2 cells were treated with a 100 μM 1:1 mixture of FFA (oleic acid and linoleic acid) for an additional 24 hours, when cells were fixed and lipids were stained using ORO. Upon counterstaining with DAPI (blue), nuclei of Cherry-tagged macroH2A1.1 and macroH2A1.2 transfected cells were observed in pink/orange (Figures 2A and B). Morphometric evaluation of cytoplasmic ORO staining showed that macroH2A1.1-overexpressing Hepa 1-6 (Figure S1, left upper panels) or HepG2 (Figure 2, right upper panels) cells were protected from lipid accumulation as compared with control cells transfected with an empty vector, while macroH2A1.2-overexpressing cells only slightly enhanced lipid content. In Hepa1-6 this trend was statistically significant in FFA-treated macroH2A1.1- or macroH2A1.2-overexpressing cells (Figure 2, lower left panel), while in HepG2 significativity was obtained for FFA-treated macroH2A1.1-overexpressing cells (Figure 2, lower right panel). Intrahepatic lipid droplets as observed in NAFLD are constituted mainly of triglycerides (TG), synthesized upon FFA intake/synthesis and cholesteryl-esters, which are instead synthesized upon augmented levels of free cholesterol. MacroH2A1.1-overexpressing Hepa1-6 (Figure 1D, left panel) or HepG2 (Figure 1D, right panel) cells consistently displayed a decreased content of TG, compared to macroH2A1.2-overexpressing and control cells. As for cholesterol content, macroH2A1.1-overexpressing Hepa1-6 and HepG2 cells showed a lower content when compared to control cells upon FFA exposure (Figure 1E). MacroH2A1.1 overexpressing HepG2 cells showed lower levels of cholesterol also in absence of FFA (Figure 1E). These data demonstrate for the first time that metabolite-binding histone variant macroH2A1.1, but not macroH2A1.2, protects against hepatic lipid accumulation *in vitro*.

**Table I:** % of Hepa 1-6 cells gated by Flow Cytometry

Cell cycle phase	G0/G1	S	G2/M
Control (empty vector)	57.5 ± 3	18.7 ± 0.7	22.2 ± 0.2
macroH2A1.1 - Cherry	56 ± 0.8	19.3 ± 0.5	21.4 ± 2.5
macroH2A1.2 - Cherry	57.1 ± 1.9	18.1 ± 0.7	22.8 ± 0.2

### MacroH2A1.1, but not macroH2A1.2, increases glycogen synthesis and glucose uptake in Hepa1-6 and HepG2 cells

**Figure 3:**
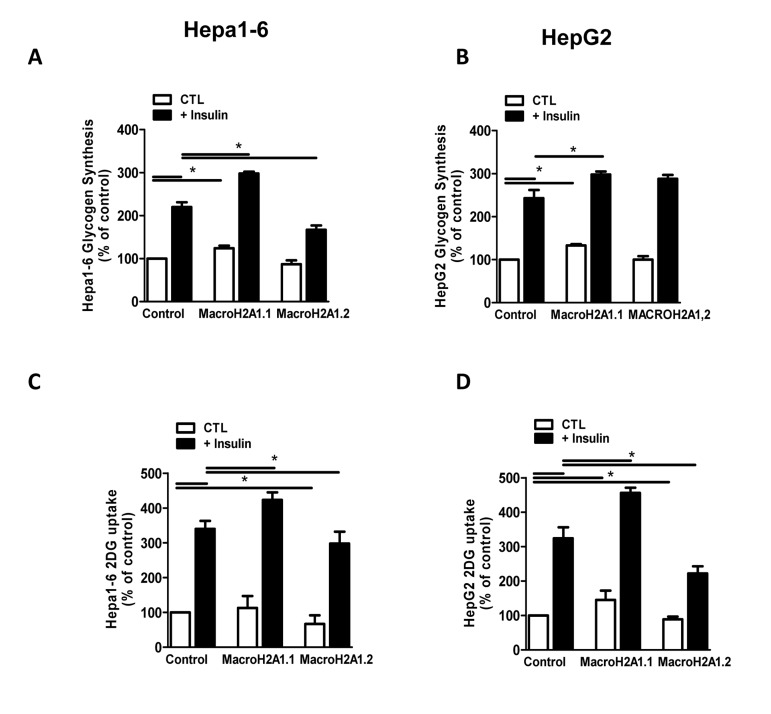
Overexpression of macroH2A1 isoforms (macroH2A1.1 or macroH2A1.2) and glycogen synthesis (**A**) and glucose uptake (B) content in Hepa1-6 and HepG2 cells. **A, B**: cells were transiently transfected as described in the legends of Figure 2. Glycogen content (**A**) and glucose uptake (**B**) were assessed by stimulation with 10^−7^ mol/L insulin, using [3-3H]-glucose incorporation and 2-deoxy-D-[2,6-3H]-glucose uptake, respectively. Results are expressed as percentage of controls, means ± SEM of four independent experiments. *p<0.05.

Although there are a few exceptions, generally insulin resistance is believed to be the primary cause of NAFLD [4]: insulin activates lipogenesis, accelerating FA synthesis and TG accumulation in the liver [28]. To determine if macroH2A1 isoforms do affect the hepatocyte response to insulin we analysed two insulin-dependent mechanisms resulting in glucose storage and transport: glycogen synthesis and glucose uptake, respectively. To this purpose, we transiently transfected Hepa1-6 and HepG2 with macroH2A1.1 or macroH2A1.2 constructs. As shown in Figure 3A, consistent with previous data [26], insulin treatment (10^−7^ M) induced an increase in glycogen storage in both cell lines. Strikingly, macroH2A1.1 overexpressing Hepa1-6 and HepG2 cells displayed an increase in glycogen content both in the absence and in the presence of insulin stimulation, while macroH2A1.2 overexpression induced a decrease in glycogen synthesis specifically in Hepa1-6 but not in HepG2 cells, where overexpression of macroH2A1.2 did not have an effect (Figure 3A and B). Measuring glucose uptake in Hepa1-6 and HepG2 cells that over-express either macroH2A1.1 or macroH2A1.2 we found that insulin-dependent glucose uptake was significantly enhanced in presence of macroH2A1.1 overexpression in both cell lines. Conversely, overexpression of macro H2A1.2 inhibited glucose uptake (Figure 3C and D). Altogether these data show that macroH2A1.1, but not macroH2A1.2 renders hepatocytes more sensitive to insulin, increasing glucose uptake and storage of glycogen, consistent with its effect on lipid accumulation (Figure 1 and 2).

### Control of genes involved in FA metabolism and of membrane lipid composition by macroH2A1 isoforms

**Figure 4:**
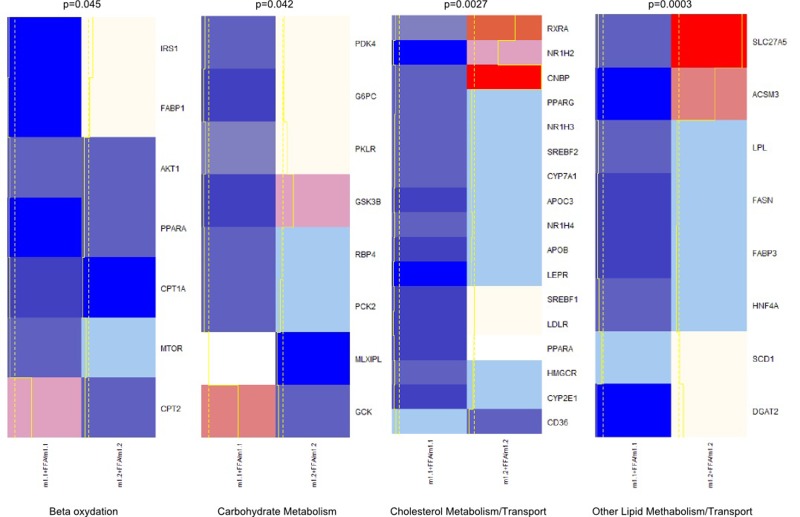
Heatmap and clusters of gene expression of Hepa1-6 cells overexpressing macroH2A1.1 (m1.1) or macroH2A1.2 (m1.2) and treated with FFA. Results are expressed as ratio of FFA-treated versus untreated cells. Optimal clusters have been computed by the pvclust method. Results were grouped in four functional processes (carbohydrate metabolism, beta-oxidation, lipid metabolism, cholesterol transport). Significance levels have been calculated via multiscale bootstrap resampling. The lower p-value of a cluster, the stronger the support of the data for the cluster. Expression levels are represented in a color scale from blue (low expressed) to red (highly expressed) (top left).

We examined the expression patterns associated with macroH2A1.1 or macroH2A1.2 over-expression of genes that may be involved in altered lipid and glucose metabolism in HepG2 and Hepa1-6 cells using a fatty liver array profiling the expression of, respectively, 84 and 81 key genes involved in four key processes implicated in the development of NAFLD: carbohydrate metabolism, beta-oxidation, lipid metabolism/transport, and cholesterol metabolism transport. Both cell types were transfected with macroH2A1.1 or macroH2A1.2 constructs and treated with FFA. We then inferred the best clustering from our qRT-PCR gene expression data by using a multiscale bootstrap resampling: this partitioning method ensures the representation of the most significant differences between genes belonging to different clusters (Figure 4 and S1). Hepa1-6 exhibit a greater variability in gene expression when overexpressing macroH2A1.1 or macroH2A1.2 in the presence of FFA, as compared to HepG2 cells (Figure 4 and S1).

**Figure 5:**
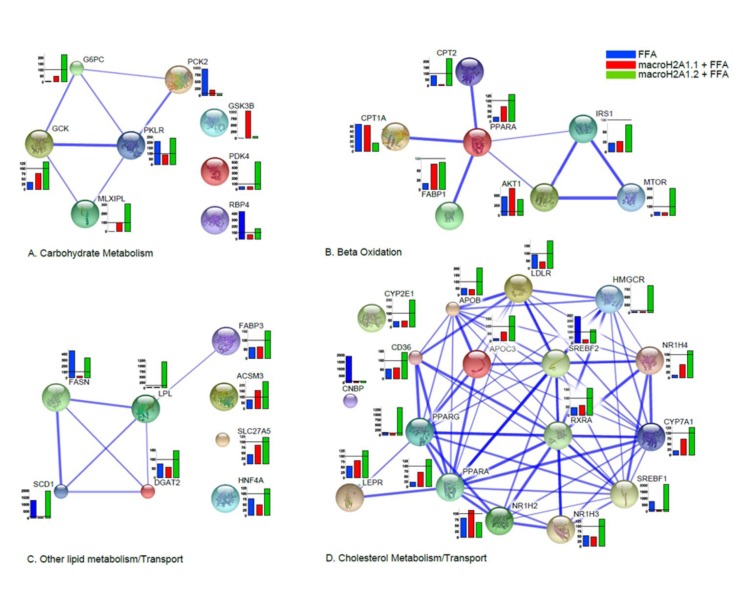
Differential effects of macroH2A1.1 and macroH2A1.2 on the expression of genes involved in lipid and carbohydrate metabolism in Hepa1-6 cells. 84 genes contained in a commercially available fatty liver array were measured by qRT-PCR in Hepa1-6 cells transiently transfected and treated with FFA, as described in the legends of Figures 1 and 2. Results were clustered in four functional processes (carbohydrate metabolism, **A**; beta-oxidation, **B**; lipid metabolism, **C**; cholesterol transport, **D**), built on a number of complementary system analyses of biological pathways (see Supplemental Materials and Methods). Results of gene expression in each histogram are represented as % of the FFA-treated mock-transfected (blue), FFA-treated macroH2A1.1-overexpressing (green) or FFA-treated macroH2A1.2 – overexpressing (red) condition related to their respective untreated controls. Results are expressed as percentage of controls, means ± SEM of two independent experiments. *p<0.05.

Moreover, data were clustered in undirected graphs, representing the above mentioned four processes of carbohydrate metabolism, beta-oxidation, lipid metabolism/transport, and cholesterol metabolism transport, where the thickness of the edges between genes correspond to the different degree of reliability of interaction, based on a number of heterogeneous data sources (protein domains, co-expression, co-localization, genetic interactions, pathways, physical and predicted interactions) (Figure 5A-D, S2A-D). In Hepa1-6, macroH2A1.1 over-expression induced, upon FFA treatment, dramatic changes in the expression of genes involved in carbohydrate metabolism when compared to macroH2A1.2 (downregulation of *G6PC, GCK, MLXIPL, PDK4* and *RBP4* and upregulation of *PCK2* and *GSK3B*) or to FFA condition (upregulation of *G6PC, GCK, MLXIPL* and *GSK3B* and down-regulation of *PCK2, PKLR and RBP4*) (Figure 5A).

In Hepa1-6 cells, macroH2A1.1 or macroH2A1.2 overexpression altered in opposite directions the expression of carnitine palmitoyltransferases, and mTOR, among others, in the beta-oxidation pathway (Figure 5B). Hepa1-6 overexpressing macroH2A1.1 also displayed a decreased expression of genes involved in fatty acid synthesis/transport (*SCD1, FASN, LPL* among others) and of genes involved in the metabolism and transport of cholesterol (*CYP2E1, CD36, PPARG, SREBF1, APOB, LDLR, HMGCR* and *SREBF2*, among others) when compared to overexpressing macroH2A1.2, upon FFA treatment (Figure 5C and D). In contrast to the dramatic changes observed in Hepa1-6 cells, macroH2A1.1 overexpressing HepG2 cells with downregulation of *RBP4, FASN, LPL* and *SCD1* displayed very few changes in gene expression when compared to macroH2A1.2 overexpressing cells, a result similar to what was observed with Hepa1-6 cells (Figure S2A-D). Therefore, one can conclude that macroH2A1.1 and trigger a distinct pattern of expression of genes involved in lipid metabolism. It is worth noting that two key genes found downregulated upon macroH2A1.1 compared to macroH2A1.2 overexpression in both cell types analyzed are stearoyl-CoA desaturase 1 (*SCD1*) and fatty acid synthase (*FASN*), enzymes that catalyze rate-limiting steps in the desaturation of FA and in the *de novo* biogenesis of FA, respectively, and shift lipid turnover towards increased lipogenesis in the elderly [29]. We thus hypothesized that macroH2A1 isoforms could differently modulate cellular membrane lipid composition: macroH2A1.1 or macroH2A1.2 were overexpressed by transient transfection in Hepa1-6 and HepG2 cells, and subsequently treated with FFA. Cell pellets were then processed for thin layer chromatography (TLC) and transesterification procedures to identify levels of total unsaturated fatty acids (UFA) [30]. As shown in Figure S3, in both HepG2 and Hepa1-6 cells macroH2A1.1 induced a decrease (~60 and 50%, respectively) in UFA content when compared with their respective untreated control cells, while macroH2A1.2 only slightly decreased the levels of UFA in HepG2 but increased them of about two fold in Hepa1-6 cells. These data demonstrate that macroH2A1.1 consistently decreases the formation of membrane UFA in the two hepatoma cell lines.

## DISCUSSION

In this study we report that the two exon splicing isoforms of macroH2A1 (macroH2A1.1 and macroH2A1.2), a histone variant functioning as a gatekeeper of cell fate and proliferation in various cell types [9-11, 31, 32], have opposite effects on the development of fat accumulation in hepatocytes. When compared with macroH2A1.2, macroH2A1.1 has a subtle structural difference that determines its tight binding to NAD+ metabolites produced by PARP1 and SIRT1 [14], two crucial enzymes involved in healthspan [33, 34]. We previously found that in the liver of mice models with NAFLD, macroH2A1.2 protein is upregulated whereas macroH2A1.1 levels do not change [21]. In the current study we found that in both human and murine cell lines (HepG2 and Hepa1-6, respectively) macroH2A1.1 overexpression, but not macroH2A1.2, protected from triglyceride and cholesterol accumulation, and sensitized cells to the action of insulin, inducing glucose uptake and gluconeogenesis. The observed slightly detrimental effect of macroH2A1.2 overexpression on lipid and glucose metabolism in vitro is consistent with the upregulation of macroH2A1.2 observed in vivo [21]; however it is not known if this upregulation of macroH2A1.2 observed in the livers of mice models of NAFLD is consequent to lipid accumulation or to other disease factors (oxidative stress, inflammation) that in turn could trigger NAFLD causatively through macroH2A1.2 upregulation. Further studies are warranted to elucidate the role of macroH2A1.2 in the “multi-hit” origin of NAFLD development. Recent advances in our understanding of the mechanisms regulating macroH2A1 alternative splicing [18, 35] are not helpful to understand in response to which stimuli the protein levels of the isoforms change in the liver. In fact mRNA levels for both macroH2A1.1 and macroH2A1.2 in NAFLD mice models and in liver biopsies from patients were variable and did not reflect the differences observed in the protein levels found in NAFLD and HCC [18, 21]. NAFLD is a serious risk factor for the development of advanced liver injury, such as steatohepatitis and HCC. MacroH2A1.1 but not macroH2A1.2 protects against the occurrence of various human cancers, influencing pathogenesis and/or survival [19, 20, 23]. Whether this is true for NAFLD-associated HCC remains to be elucidated. Our gene expression analysis, covering a large array of players involved in lipid metabolism, showed dramatic changes in Hepa1-6 overexpressing macroH2A1.1 as compared to macroH2A1.2 overexpressing cells in the presence of FFA, which were less accentuated in HepG2 cells. These profound changes in Hepa1-6 corroborate an anti-lipidogenic role for macroH2A1.1 as compared to macroH2A1.2. It remains to be explained why on the one hand similar effects of macroH2A1 isoforms on lipid accumulation and glucose metabolism were observed in Hepa1-6 and HepG2 cells, while on the other hand a different magnitude of impact on gene expression was observed in the two cell types. This could likely reflect species-specific (mouse *versus* human) and different levels of endogenous macroH2A1 isoforms expression (Figure 1) between the two cell models. Nevertheless, upon FFA treatment we observed a downregulation of *SCD1* and *FASN* upon macroH2A1.1- as compared to macroH2A1.2-overexpression, in both Hepa1-6 and HepG2 cells. Low levels of *SCD1* and *FASN* expression are protective against obesity, insulin resistance and NAFLD [36, 37]. Our membrane lipid profile assay showed an overall increase in membrane unsaturated FA, which could be related functionally to an increasing expression/activity of these enzymes. The profound differences, caused by a few amino acid discrepancy between macroH2A1.1 and macroH2A1.2 isoforms, in gene expression and lipid metabolism in the liver are remarkable. In this respect, the property whereby macroH2A1.1 binds with very tight affinity with OAADPR produced by SIRT1 is intriguing, considering that the activation of these enzymes is considered protective against NAFLD favouring a overall healthy aging [38], and is under scrutiny for drug design [39, 40].

Interestingly, OAADPR can be bound and deacetylated by the macrodomain contained in proteins displaying different functions and participating to distinct pathways other than macroH2A1, suggesting an evolutionary conserved molecular affinity [41]. OAADPR and macroH2A1 have been independently reported to regulate gene silencing and the gating of members of the large superfamily of transient receptor potential (TRP) ion channels [42, 43]. Of note, macroH2A1.1 suppresses growth of cancer cells in a manner dependent on its ability to bind NAD+ metabolites such as OAADPR [18]. Whether this property plays a role in liver diseases and more broadly in healthy aging is unknown. The presence of a metabolite-binding function in a chromatin component opens new potential connections between gene expression and lipid metabolism in the liver, which warrants further structural and functional studies. Macro domains could also represent attractive and novel drug targets, likely in connection to the well-established SIRT1 pathway.

## METHODS

### Cell cultures, constructs, transfection, treatment and imaging

Both Hepa1-6 and HepG2 hepatoma cells (of mouse and human origin, respectively) were cultured in Dulbecco's MEM supplemented with 10% fetal bovine serum (FBS), 2mM L-Glutamine and 100U penicillin/streptomycin mix as previously described [26,27]. Cells were passaged every 3-4 days and cultured in 6 wells plates for imaging experiments. For treatment with free fatty acids, cells were exposed 24 hours to a mixture of albumin conjugated oleic acid and linoleic acid (OA/LA, Sigma) to a final concentration of 100 μM. Constructs for macroH2A1.1 and macroH2A1.2 were previously described [16, 44]. Transient transfections were performed using Lipofectamine reagent (Invitrogen), according to manufacturer's instructions. Oil Red O (ORO) staining of lipid droplets was performed as previously described [26]. Fluorescent images were taken on a Nikon Eclipse TE200 inverted fluorescent microscope, using a U-III advanced exposure system with Multi-point sensor, and a Nikon FDX-35 camera. Filters used were i) G-2A; Ex510-560; DM 575; BA590 (Red); ii) UV-2A; Ex 330-380; DM 400; BA 420 (Dapi): Dapi-Fitc-Rhodamine (Cherry and merge). Software ImageJ (NIH) was used to quantify ORO-stained lipid droplets in individual Hepa1-6 or HepG2 cells [26].

### Cell cycle analysis

Cells were harvested at least 3 hours before the experiment and fixed with 1ml of 70% cold ethanol at −20°C, as indicated by the Muse Cell Cycle Kit User's Guide. 200 μl of ethanol-fixed cells were washed in PBS and stained with the Muse Cell Cycle Reagent containing propidium iodide and RNAse A for 30 minutes at room temperature, before loading on Muse Cell Analyzer (Millipore, Italy) according to the supplied staining protocol.

### Histone acid extraction and immunoblotting from cultured cells

Histone fraction was enriched using an acid extraction protocol. Briefly, cell pellets were suspended and homogenized in 200 μl of H-lysis solution (0.2 M sucrose, 3 mM CaCl2, 1 mM Tris-HCl pH8.0, 0.5 NP40, protease inhibitor cocktail), incubated on ice for 8 min, and centrifuged at 1,300×g, 4°C, for 5 min to separate supernatant from nuclei fraction (P1). P1 was washed once with H-wash solution (300 mM NaCl, 5 mg MgCl2, 5 mM DTT, 0.5% NP40) and lysed for 30 min in 100 μl H-extract solution (0.5 mM HCl, 10% glycerol, protease inhibitor cocktail), followed by centrifugation at 13,000×g 4°C, for 5 min. Finally, TCA precipitation was performed. Equal amounts of protein were separated by SDS-PAGE, transferred to PVDF membrane (Amersham, Buckinghamshire, UK) and the resulting immune-complex was visualized using the molecular imager ChemiDoc XRS + system (Bio-Rad). Densitometry was performed using Image Lab Software (Bio-Rad). Primary antibodies for MacroH2A1.1 and MacroH2A1.2 were generated at the European Molecular Biology Laboratory (EMBL) and were a courtesy of Prof. Andreas Ladurner (Ludwig Maximilian University, LMU, Munich, Germany). Antibodies against histone H2A (Cell Signaling) were used to normalize protein levels.

### Triglyceride and cholesterol measurement in cell cultures

Lipids were extracted as previously described [26]. Intracellular triglycerides (TG) were quantified with the commercially available triglyceride glycerol phosphate oxidase-paminophenazone (GPO-PAP) kit (Roche), while cholesterol was detected using the cholesterol quantitation kit (Calbiochem), respectively.

### Glycogen synthesis and glucose uptake assays

Glycogen synthesis in Hepa1-6 and HepG2 cells was assessed by measuring [3-3H]-glucose incorporation per microgram of protein as previously described [45]. Measurements of 2-deoxy-D-[2,6-3H]-glucose uptake by Hepa1-6 and HepG2 cells were performed as previously described [46]. Non-specific glucose uptake was determined in the presence of 10 mM cytochalasin B. Cell-associated radioactivity was measured by liquid scintillation counting in a β-counter (Wallac 1409, Perkin Elmer).

### RNA extraction and Fatty Liver Array profile

Total RNA was isolated using the RNeasy Mini Kit (Qiagen, Milan, Italy) and subsequently treated with deoxyribonuclease I. Human and Fatty Liver RT^2^ Profiler™ PCR Array profiles were then assessed following the manufactures protocol (SABioscience, Milan, Italy). Reactions were set up in 96-well plates using a 7700HT Real-Time PCR System (Applied Biosystems, Foster City, CA), Optical data obtained were analyzed using the default and variable parameters available in the SDS software package (version 1.9.1; Applied Biosystems, Foster City, CA).

### System biology analyses of gene expression

Heatmaps and clusters have been calculated and drawn by R framework, ver. 2.15.2. Optimal clusters have been computed by pvclust [47], an R package for assessing the uncertainty in hierarchical cluster analysis. For each cluster in hierarchical clustering, significance levels have been calculated via multiscale bootstrap resampling. The lower p-value of a cluster, the stronger the support of the data to the cluster. Interactions between genes participating to the carbohydrate metabolism, beta-oxidation, cholesterol metabolism/transport and other lipid metabolism/transport processes were reconstructed both for Hepa1-6 and HepG2 cells. Four hypergraphs per cell type were then built on a number of heterogeneous data sources: protein domains (Interpro and PFAM), co-expression (curated literature and Gene Expression Omnibus), co-localization (Gene Expression Omnibus), genetic interactions (BIOGRID and IREF), pathways (PathwayCommons, IMID, NCI NATURE, REACTOME, KEGG and BIOCARTA), physical interactions (BIOGRID, BIND, HPRD, INTACT, MINT, MPPI and OPHID) and predicted interactions (curated literature). Any two genes were connected by an edge, whenever at least an interaction evidence of any of the abovementioned interaction categories was found. Several pairs of genes resulted to be connected by more than one edge. Each hypergraph was weighted and signed. Weights over the edges represented the reliability of the corresponding interactions and were proportional to the thickness of the edges. Gene expressions for cells transfected with macroH2A1.1 or macroH2A1.2 constructs with or without exposure to FAs were represented with histograms. Each hypergraph was deterministically transformed into an undirected graph by applying an injective function to the sets of weights over the edges.

The function 
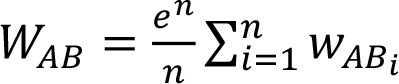
 where n is the number of edges connecting any two nodes *A* and *B* and *i* refers to the i^th^ edge, takes the weights of the edges connecting any two nodes in input and gives a unique value in output. Constitutively, it gives more and more importance to the genes that are connected by multiple edges.

### Lipidomic

Cells were thoroughly washed with phosphate buffer, added with water and pelleted by centrifugation at 14,000 g for 40 min at 4°C. The pellet was resuspended in pure water and centrifuged, then was dissolved in 2:1 chloroform:methanol and examined by thin layer chromatography (TLC using a bidimensional system; first eluent: chloroform: methanol:acetic acid:water 55:33:9:3; second eluent: hexane:diethyl ether:acetic acid 30:29:1) to determine the purity of the phospholipid fraction. The phospholipid extract was then treated with 0.5 M KOH/MeOH for 10 min at room temperature to convert the fatty acid residues of the phospholipids into their corresponding fatty acid methyl esters (FAMEs). After this transesterification step, FAMEs were extracted with n-hexane, and analyzed by gas chromatography. Geometrical trans unsaturated fatty acids were identified by comparison with standard references obtained by synthesis, as described [30].

### Statistical analysis

Results are expressed as means ± SEM. Comparisons between groups were performed using a Student's t-test with non-parametric Mann-Whitney test using GraphPad Prism Software (version 5.00 for Windows, San Diego, CA, USA): a p-value ≤ 0.05 was considered significant.

## SUPPLEMENTAL FIGURES



## Abbreviations

NAFLD: nonalcoholic-fatty-liver-disease
NASH: nonalcoholic steatohepatitis
TG: triglycerides
TC: total cholesterol
HCC: hepatocellular carcinoma
ORO: Oil red O
FFA: free fatty acids
UFA: unsaturated fatty acids
FA: fatty acids
