# Supplementary material for: SIRT1-metabolite binding histone macroH2A1.1 protects hepatocytes against lipid accumulation
Source: Aging (Albany NY). 2014 Jan 28;6(1):35–47. doi: 10.18632/aging.100632 (PMC3927808; doi:10.18632/aging.100632)
Supplement: Supplementary file 1 [file aging-06-035-s001.pdf]

46. Deblon N, Bourgoin L, Veyrat-Durebex C, Peyrou M, Vinciguerra M, Caillon A, Maeder C, Fournier M, Montet X, Rohner-Jeanrenaud F, Foti, M. Chronic mTOR inhibition by rapamycin induces muscle insulin resistance despite weight loss in rats. Br J Pharmacol. 2012. 165:2325-2340.
47. Suzuki R, Shimodaira H: Pvclust. an R package for assessing the uncertainty in hierarchical clustering. Bioinformatics. 2006. 22:1540-1542.

SUPPLEMENTAL FIGURES

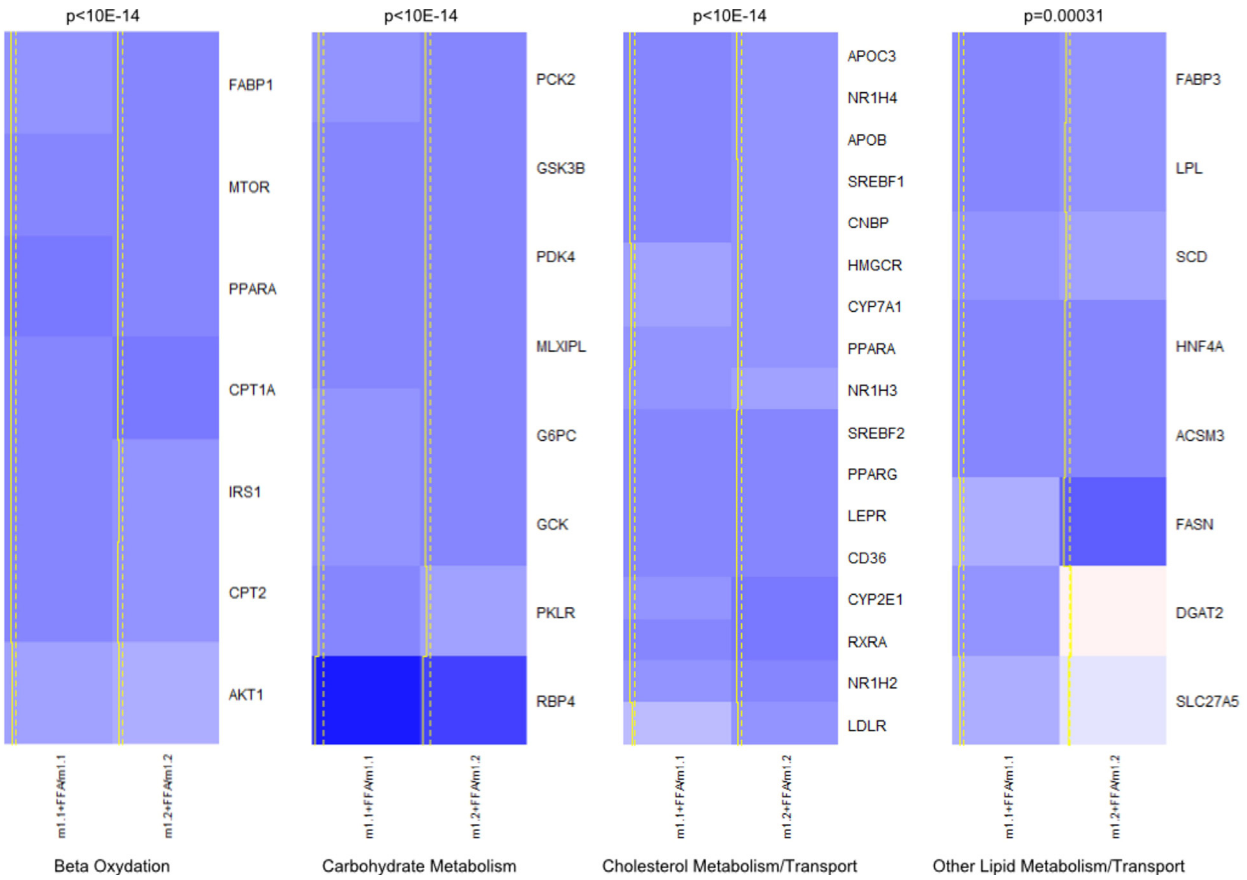

Supplemental Figure 1. Heatmap and clusters of gene expression of HepG2 cells overexpressing macroH2A1.1 (m1.1) or macroH2A1.2 (m1.2) and treated with FFA. Results are expressed as ratio of FFA-treated versus untreated cells. Optimal clusters have been computed by the pvclust method. Results were grouped in four functional processes (carbohydrate metabolism, beta-oxidation, lipid metabolism, cholesterol transport). Significance levels have been calculated via multiscale bootstrap resampling. The lower p-value of a cluster, the stronger the support of the data to the cluster. Expression levels are represented in a colour scale from blue (low expressed) to red (highly expressed) (top left).

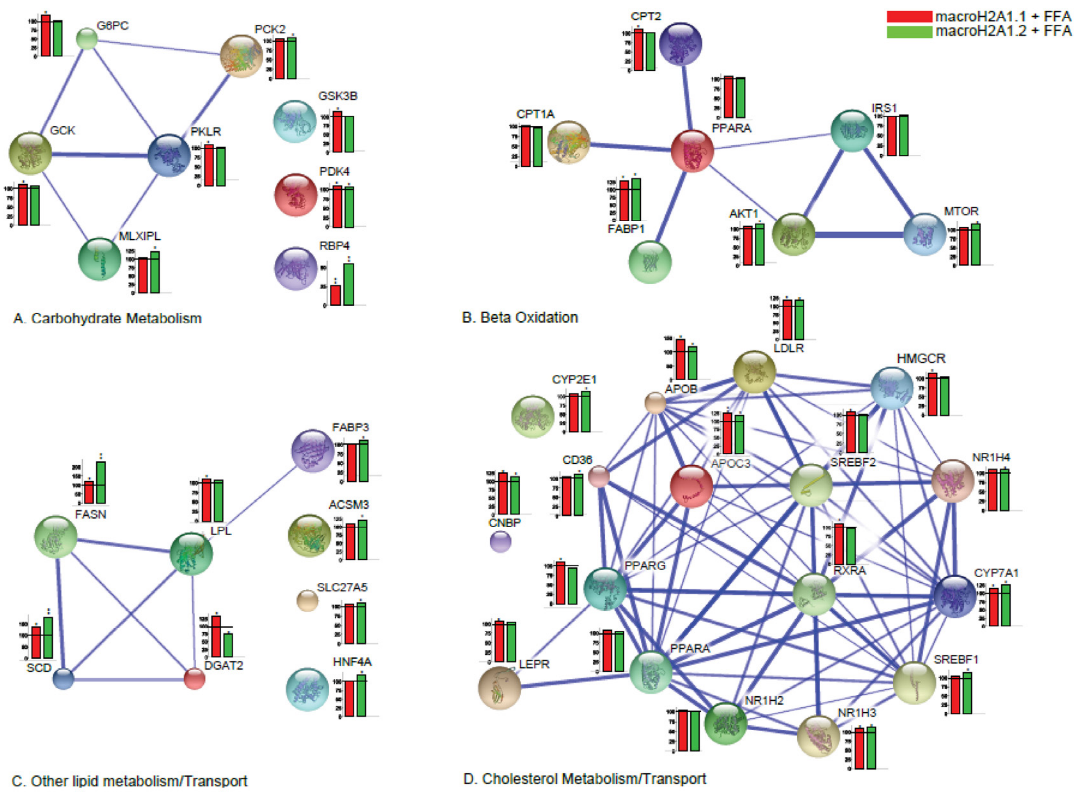

Supplemental Figure 2. Differential effects of macroH2A1.1 and macroH2A1.2 on the expression of genes involved in lipid and carbohydrate metabolism in HepG2 cells. 81 genes contained in a commercially available fatty liver array were measured by qRT-PCR in HepG2 cells transiently transfected and treated with FFA, as described in the legends of Figure 1 and 2. Results were clustered in four functional hypergraphs (carbohydrate metabolism, **A**; beta-oxidation, **B**; lipid metabolism/transport, **C**; cholesterol transport/metabolism, **D**), built on a number of complementary system analyses of biological pathways, as described in the Supplemental Material & Methods section. Results of gene expression in each histogram are represented as % of FFA-treated mock-transfected (blue), FFA-treated macroH2A1.1-overexpressing (green) or FFA-treated macroH2A1.2 – overexpressing (red) condition related to their respective untreated controls. Results are expressed as percentage of controls, means ± SEM of two independent experiments. *p<0.05.

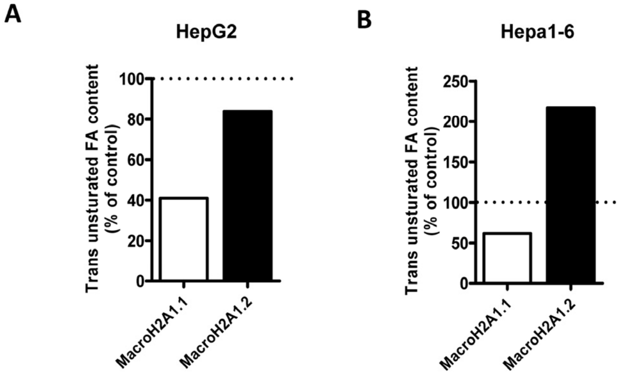

Supplemental Figure 3. Overexpression of macroH2A1 isoforms (macroH2A1.1 or macroH2A1.2) and trans unsaturated fatty acids in Hepa1-6 and HepG2 cells. A, B: cells were transiently transfected and treated with FFA as described in the legends of Figure 1 and 2. Trans unsaturated fatty acids content was measured using TLC-chromatography in HepG2 (**A**) and Hepa1-6 (**B**) upon FFA treatment. Results are expressed as percentage of respective controls (untreated macroH2A1.1- or macroH2A1.2-overexpressing cells), and are means of two independent experiments.
